# 2-(4-Meth­oxy­phen­yl)-4*H*-1,3,2-benzoxathia­phosphinine 2-sulfide

**DOI:** 10.1107/S1600536810053146

**Published:** 2011-01-15

**Authors:** Vitaly A. Osyanin, Elena A. Ivleva, Victor B. Rybakov, Yurij N. Klimochkin

**Affiliations:** a Samara State Technical University, Molodogvardeyskay Str. 244, 443100 Samara, Russian Federation; b Department of Chemistry, Moscow State University, 119992 Moscow, Russian Federation

## Abstract

The asymmetric unit of the title compound, C_14_H_13_O_2_PS_2_, contains two crystallographically independent mol­ecules, which differ in the conformation of the 1,3,2-benzoxathia­phosphinine moieties (screw boat in the first mol­ecule and envelope in the second mol­ecule). In the crystal, neither classical nor non-classical hydrogen bonds are found. Weak inter­actions (about 2.9–3.0 Å) between the lone pair of the terminal S atoms with H atoms occur. This compound was further characterized by ^1^H NMR and IR spectroscopy.

## Related literature

Lawesson’s reagent is widely used for transformation of a carbonyl functional group into a thio­carbonyl, see: Ozturk *et al.* (2007 ▶). Lawesson’s reagent reacts with 1,2-naphtho­quinone-1-methide precursors to give 1*H*-naphtho­[1,2-*e*][1,3,2]oxathia­phosphinine 2-sulfide derivatives, which are of inter­est as herbicides, see: El-Kateb & El-Rahman (2006 ▶); El-Kateb *et al.* (1991 ▶); Maigali *et al.* (2009 ▶). For conformational calculations, see: Cremer & Pople (1975 ▶); Zefirov *et al.* (1990 ▶); Zotov *et al.* (1997 ▶). For a description of the Cambridge Structural Database, see: Allen (2002 ▶).
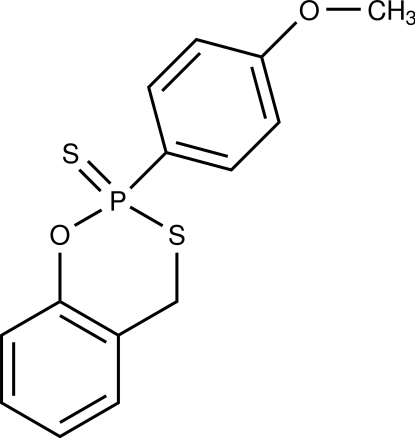


## Experimental

### 

#### Crystal data

C_14_H_13_O_2_PS_2_
*M* *_r_* = 308.35
Triclinic, 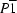

*a* = 10.0548 (5) Å
*b* = 10.0804 (5) Å
*c* = 14.8913 (7) Å
α = 94.322 (4)°
β = 91.121 (4)°
γ = 111.675 (4)°
*V* = 1396.79 (12) Å^3^
*Z* = 4
Cu *K*α radiation
μ = 4.50 mm^−1^
*T* = 150 K
0.20 × 0.14 × 0.05 mm

#### Data collection

Oxford Diffraction Xcalibur Atlas Gemini ultra diffractometer
Absorption correction: analytical [*CrysAlis PRO* (Oxford Diffraction, 2010 ▶); based on expressions derived by Clark & Reid (1995 ▶)] *T*_min_ = 0.499, *T*_max_ = 0.816
27233 measured reflections
4926 independent reflections
4175 reflections with *I* > 2σ(*I*)
*R*_int_ = 0.051

#### Refinement

*R*[*F* ^2^ > 2σ(*F* ^2^)] = 0.031
*wR*(*F* ^2^) = 0.078
*S* = 1.05
4926 reflections
345 parameters
H-atom parameters constrained
Δρ_max_ = 0.38 e Å^−3^
Δρ_min_ = −0.28 e Å^−3^

### 

Data collection: *CrysAlis PRO* (Oxford Diffraction, 2010 ▶); cell refinement: *CrysAlis PRO*; data reduction: *CrysAlis PRO*; program(s) used to solve structure: *OLEX2* (Dolomanov *et al.*, 2009 ▶); program(s) used to refine structure: *SHELXL97* (Sheldrick, 2008 ▶); molecular graphics: *ORTEP-3* (Farrugia, 1997 ▶); software used to prepare material for publication: *OLEX2*.

## Supplementary Material

Crystal structure: contains datablocks global, I. DOI: 10.1107/S1600536810053146/vm2065sup1.cif

Structure factors: contains datablocks I. DOI: 10.1107/S1600536810053146/vm2065Isup2.hkl

Additional supplementary materials:  crystallographic information; 3D view; checkCIF report

## Figures and Tables

**Fig. 1.**
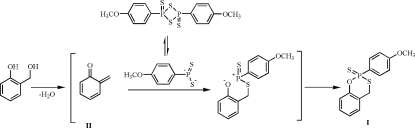
Synthesis of the title compound.

**Fig. 2.**
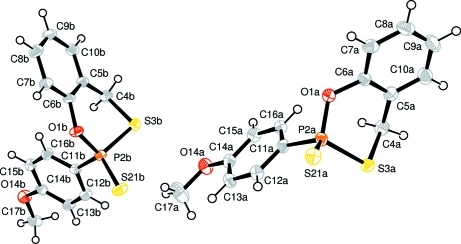
ORTEP-3 (Farrugia, 1997) plot of molecular structure of the title compound showing the atom-numbering scheme. Thermal displacement ellipsoids are drawn at the 50% probability level. H atoms are presented as a small spheres of arbitrary radius.

## supplementary crystallographic information

### Comment

Lawesson's reagent is widely used for transformation of a carbonyl functional group into a thiocarbonyl (Ozturk *et al.*, 2007). At the same time, the reaction of Lawesson's reagent with compounds having two nucleophilic or one nucleophilic and one electrophilic centers may lead to heterocyclic rings incorporating part of Lawesson's reagent.

It was shown that Lawesson's reagent reacts with 1,2-naphthoquinone-1-methide precursors to give 1*H*-naphtho[1,2-e][1,3,2]oxathiaphosphinine 2-sulfide derivatives which are interesting as herbicides (El-Kateb & El-Rahman, 2006; El-Kateb *et al.*, 1991; Maigali *et al.*, 2009). However, preparation of 4*H*-1,3,2-benzoxathiaphosphinine 2-sulfides from salicylic alcohols was not described in literature. The 2-(4-methoxyphenyl)-4*H*-1,3,2-benzoxathiaphosphinine 2-sulfide was prepared from Lawesson's reagent and *o*-hydroxybenzyl alcohol in *o*-xylene at reflux in 35% yield. A mechanism accounting for the formation of structure I is depicted in Fig. 1. The hydroxybenzyl alcohol loses a molecule of water to give the *o*-quinone methide II. A nucleophilic attack on the methylene group of II by the sulfur anion of the monomeric form of Lawesson's reagent produces the zwitterionic intermediate which is cyclized to give the end product I.

The asymmetric unit of a crystal of I contains two crystallographically independent molecules, which are different by the conformation of the 1,3,2-benzoxathiaphosphinine moieties: in molecule a - screw boat, and in molecule b - distorted envelope. The Zotov-Palyulin puckering parameters for molecule a are: *S* = 0.886, θ = 80.83°, ψ_2_ = 347.94°, σ = 4.21 (Zefirov *et al.*, 1990; Zotov *et al.*, 1997). Cremer-Pople parameters for comparison: *Q* = 0.750 Å, θ = 76.92°, φ_2_ = 341.43° (Cremer & Pople, 1975). For molecule b: *S* = 0.751, θ = 35.92°, ψ_2_ = 356.11°, σ = 3.86 (Zotov-Palyulin), and *Q* = 0.666 Å, θ = 52.87°, φ_2_ = 2.08° (Cremer-Pople).

In the crystal structure neither classical nor non-classical hydrogen bonds are found, but weak interactions between lone pairs of terminal S atoms with H atoms are found: C12*a*–H12*a*···S21*a* [C12*a*–H12*a* = 0.95 Å, C12*a*···S21*a* = 3.379 (2) Å, H12*a*···S21*a* = 2.894 Å, angle C12*a*–H12*a*···S21*a* = 113°]; C17*a*–H17*b*···S21*b*Å [C17*a*–H17*b* = 0.98 Å, C17*a*···S21*b* = 3.817 (3) Å, H17*b*···S21*b* = 2.847 Å, angle C17*a*–H17*b*···S21*b* = 170°]; C17*a*–H17*c*···S21*a*^i^ [C17*a*–H17*c* = 0.98 Å, C17*a*···S21*a*^i^ = 3.906 (3) Å, H17*c*···S21*a*^i^ = 2.978 Å, angle C17*a*–H17*c*···S21*a*^i^ = 159°]; C4b–H4*b*2···S3a^ii^ [C4b–H4*b*2 = 0.99 Å, C4b···S3a^ii^ = 3.796 (2) Å, H4*b*2···S3a^ii^ = 2.996 Å, angle C4b–H4*b*2···S3a^ii^ = 139°]; C8b–H8b···S21*b*^iii^ [C8b–H8b = 0.95 Å, C8b···S21*b*^iii^ = 3.657 (3) Å, H8b···S21*b*^iii^ = 2.944 Å, angle C8b–H8b···S21*b*^iii^ = 133°]. Symmetry codes: (i) -*x* + 1, -*y* + 1, -*z*; (ii) *x*, *y* + 1, *z* + 1; (iii) *x*, *y* + 1, *z*.

### Experimental

A mixture of Lawesson's reagent (3.31 g, 8.2 mmol) and *o*-hydroxybenzyl alcohol (1 g, 8.2 mmol) in *o*-xylene (30 ml) was refluxed for 2 h. The solvent was removed *in vacuo* and the residue was dissolved in 15 ml of methanol at reflux and cooled to room temperature. Insoluble impurity was filtered off and filtrate then stored at 253 K overnight. The precipitate formed was then filtered, washed ice-cold methanol. Recrystallization of the crude product from methanol gave 0.88 g of colourless crystals. Yield 35%, m.p. 357-358 K. IR-spectra, ν, cm^-1^: 3067 (CH-aromatic.), 2966, 2928, 2839 (CH-aliphatic), 1593 (C═C), 1562, 1497, 1481, 1474, 1447 (P–C), 1300, 1261, 1211, 1173, 1111, 1022, 926, 833, 763, 729, 698, 683. MS(ESI): m/*z* 308 [*M*]^+^ (100), 275 [*M*-SH]^+^ (36), 243 (14), 242 (15), 169 (13), 153 (50), 139 (57), 137 [C_7_H_5_OS]^+^ (23), 122 (15). ^1^H NMR, δ: 3.82 s (3*H*, OCH_3_), 4.14-4.27 m (2*H*, CH_2_), 7.11 dd (2*H*, ^3^J = 8.86 Hz, ^4^J PH = 3.76 Hz, CH_3_OCCH), 7.18-7.21 m (2*H*, H-6,8), 7.36-7.40 m (2*H*, H-5,7), 7.90 dd (2*H*, ^3^J PH = 14.50 Hz, ^3^J = 8.86 Hz, PCCH). Anal. calc. for C_14_H_13_O_2_PS_2_, %: C 54.53; H 4.25; S 20.80. Found, %: C 54.61; H 4.21; S 20.71.

Single crystals for *X*-ray analysis were obtained by slow evaporation of a methanol solution. IR-spectrum was recorded (in KBr) on Shimadzu FTIR-8400S. Mass-spectrum was measured on Finnigan Trance DSQ spectrometer. ^1^H NMR spectrum was obtained in DMSO-d_6_ on Jeol JNM-ECX400 (400 MHz), using TMS as internal standard. Elemental composition was determined on Euro Vector EA-3000 elemental analyzer.

### Refinement

C-bound H-atoms were placed in calculated positions with C–H 0.95 Å for aromatic, 0.99 Å for methylene with *U*_iso_(H) = 1.2*U*_eq_(C) and 0.98 Å for methyl with *U*_iso_(H) = 1.5*U*_eq_(C). All H atoms refined as riding.

Technical problems during the diffraction experiment led to the loss of 87 reflections.

### Crystal data

**Table:**

C_14_H_13_O_2_PS_2_	*Z* = 4
*M**_r_* = 308.35	*F*(000) = 640
Triclinic, *P*1	*D*_x_ = 1.466 Mg m^−^^3^
Hall symbol: -P 1	Melting point = 357–358 K
*a* = 10.0548 (5) Å	Cu *K*α radiation, λ = 1.54184 Å
*b* = 10.0804 (5) Å	Cell parameters from 11905 reflections
*c* = 14.8913 (7) Å	θ = 3.0–67.2°
α = 94.322 (4)°	µ = 4.50 mm^−^^1^
β = 91.121 (4)°	*T* = 150 K
γ = 111.675 (4)°	Prism, colourless
*V* = 1396.79 (12) Å^3^	0.20 × 0.14 × 0.05 mm

### Data collection

**Table:**

Oxford Diffraction Xcalibur Atlas Gemini ultra diffractometer	4926 independent reflections
Radiation source: fine-focus sealed tube	4175 reflections with *I* > 2σ(*I*)
mirror	*R*_int_ = 0.051
ω scans	θ_max_ = 67.4°, θ_min_ = 3.0°
Absorption correction: analytical [*CrysAlis PRO* (Oxford Diffraction, 2010); based on expressions derived by Clark & Reid (1995)]	*h* = −12→12
*T*_min_ = 0.499, *T*_max_ = 0.816	*k* = −11→12
27233 measured reflections	*l* = −17→17

### Refinement

**Table:**

Refinement on *F*^2^	Primary atom site location: structure-invariant direct methods
Least-squares matrix: full	Secondary atom site location: difference Fourier map
*R*[*F*^2^ > 2σ(*F*^2^)] = 0.031	Hydrogen site location: inferred from neighbouring sites
*wR*(*F*^2^) = 0.078	H-atom parameters constrained
*S* = 1.05	*w* = 1/[σ^2^(*F*_o_^2^) + (0.0361*P*)^2^ + 0.5302*P*] where *P* = (*F*_o_^2^ + 2*F*_c_^2^)/3
4926 reflections	(Δ/σ)_max_ = 0.001
345 parameters	Δρ_max_ = 0.38 e Å^−^^3^
0 restraints	Δρ_min_ = −0.28 e Å^−^^3^

### Special details

**Table:**

Experimental. *CrysAlis Pro*(Oxford Diffraction, 2010); Analytical numeric absorption correction using a multifaceted crystal model based on expressions derived by Clark & Reid (1995).
Geometry. All s.u.'s (except the s.u. in the dihedral angle between two l.s. planes) are estimated using the full covariance matrix. The cell s.u.'s are taken into account individually in the estimation of s.u.'s in distances, angles and torsion angles; correlations between s.u.'s in cell parameters are only used when they are defined by crystal symmetry. An approximate (isotropic) treatment of cell s.u.'s is used for estimating s.u.'s involving l.s. planes.
Refinement. Refinement of F^2^ against ALL reflections. The weighted R-factor wR and goodness of fit S are based on F^2^, conventional R-factors R are based on F, with F set to zero for negative F^2^. The threshold expression of F^2^ > 2σ(F^2^) is used only for calculating R-factors(gt) etc. and is not relevant to the choice of reflections for refinement. R-factors based on F^2^ are statistically about twice as large as those based on F, and R-factors based on ALL data will be even larger.

### Fractional atomic coordinates and isotropic or equivalent isotropic displacement parameters (Å^2^)

**Table:**

	*x*	*y*	*z*	*U*_iso_*/*U*_eq_	
O1a	0.02926 (16)	0.29773 (15)	−0.14746 (10)	0.0277 (3)	
P2a	0.15135 (6)	0.24002 (6)	−0.11687 (3)	0.02317 (13)	
S3a	0.04617 (6)	0.01896 (6)	−0.12506 (4)	0.02639 (13)	
C4a	−0.1381 (2)	0.0133 (2)	−0.12138 (15)	0.0292 (5)	
H4a1	−0.2060	−0.0876	−0.1243	0.035*	
H4a2	−0.1500	0.0630	−0.0640	0.035*	
C5a	−0.1703 (2)	0.0843 (2)	−0.19912 (14)	0.0267 (5)	
C6a	−0.0826 (2)	0.2236 (2)	−0.21191 (14)	0.0250 (5)	
C7a	−0.1048 (2)	0.2957 (3)	−0.28197 (15)	0.0301 (5)	
H7a	−0.0420	0.3910	−0.2891	0.036*	
C8a	−0.2218 (3)	0.2251 (3)	−0.34208 (16)	0.0377 (6)	
H8a	−0.2404	0.2728	−0.3906	0.045*	
C9a	−0.3108 (3)	0.0866 (3)	−0.33152 (17)	0.0415 (6)	
H9a	−0.3900	0.0392	−0.3732	0.050*	
C10a	−0.2862 (3)	0.0153 (3)	−0.26080 (17)	0.0365 (6)	
H10a	−0.3481	−0.0805	−0.2543	0.044*	
S21a	0.32266 (6)	0.29002 (6)	−0.18310 (4)	0.02974 (14)	
C11a	0.1791 (2)	0.3094 (2)	−0.00060 (14)	0.0236 (4)	
C12a	0.3126 (2)	0.3441 (2)	0.04299 (14)	0.0264 (5)	
H12a	0.3898	0.3382	0.0093	0.032*	
C13a	0.3357 (2)	0.3873 (2)	0.13437 (14)	0.0268 (5)	
H13a	0.4278	0.4110	0.1631	0.032*	
C14a	0.2221 (2)	0.3953 (2)	0.18358 (14)	0.0256 (5)	
C15a	0.0881 (2)	0.3626 (2)	0.14060 (15)	0.0274 (5)	
H15a	0.0112	0.3697	0.1741	0.033*	
C16a	0.0669 (2)	0.3200 (2)	0.04959 (15)	0.0266 (5)	
H16a	−0.0248	0.2977	0.0207	0.032*	
O14a	0.23190 (17)	0.43446 (17)	0.27326 (10)	0.0320 (4)	
C17a	0.3668 (3)	0.4683 (4)	0.32061 (17)	0.0509 (7)	
H17a	0.3959	0.3856	0.3137	0.076*	
H17b	0.3582	0.4921	0.3847	0.076*	
H17c	0.4390	0.5505	0.2959	0.076*	
O1b	0.33103 (15)	0.89365 (15)	0.58572 (10)	0.0249 (3)	
P2b	0.26816 (6)	0.73662 (6)	0.62048 (4)	0.02184 (13)	
S3b	0.05104 (6)	0.66625 (6)	0.59061 (4)	0.02646 (13)	
C4b	0.0279 (2)	0.8259 (2)	0.64340 (15)	0.0277 (5)	
H4b1	−0.0733	0.8152	0.6335	0.033*	
H4b2	0.0480	0.8318	0.7092	0.033*	
C5b	0.1228 (2)	0.9638 (2)	0.60826 (13)	0.0236 (4)	
C6b	0.2616 (2)	0.9922 (2)	0.58256 (14)	0.0227 (4)	
C7b	0.3455 (2)	1.1212 (2)	0.55097 (15)	0.0275 (5)	
H7b	0.4394	1.1363	0.5323	0.033*	
C8b	0.2900 (3)	1.2277 (2)	0.54712 (16)	0.0325 (5)	
H8b	0.3464	1.3171	0.5262	0.039*	
C9b	0.1529 (3)	1.2042 (2)	0.57358 (15)	0.0316 (5)	
H9b	0.1156	1.2779	0.5717	0.038*	
C10b	0.0702 (2)	1.0739 (2)	0.60266 (14)	0.0280 (5)	
H10b	−0.0248	1.0582	0.6193	0.034*	
S21b	0.35029 (6)	0.60954 (6)	0.56361 (4)	0.03111 (14)	
C11b	0.2993 (2)	0.7683 (2)	0.74075 (14)	0.0224 (4)	
C12b	0.2885 (2)	0.6534 (2)	0.79025 (15)	0.0278 (5)	
H12b	0.2694	0.5616	0.7596	0.033*	
C13b	0.3052 (2)	0.6707 (2)	0.88353 (15)	0.0274 (5)	
H13b	0.2974	0.5916	0.9168	0.033*	
C14b	0.3335 (2)	0.8054 (2)	0.92778 (14)	0.0251 (5)	
C15b	0.3472 (2)	0.9212 (2)	0.87922 (15)	0.0286 (5)	
H15b	0.3687	1.0134	0.9099	0.034*	
C16b	0.3299 (2)	0.9033 (2)	0.78634 (15)	0.0265 (5)	
H16b	0.3387	0.9830	0.7534	0.032*	
O14b	0.34857 (18)	0.83466 (17)	1.01926 (10)	0.0333 (4)	
C17b	0.3349 (3)	0.7191 (3)	1.07348 (15)	0.0344 (5)	
H17d	0.2394	0.6441	1.0620	0.052*	
H17e	0.3481	0.7547	1.1374	0.052*	
H17f	0.4079	0.6794	1.0581	0.052*	

### Atomic displacement parameters (Å^2^)

**Table:**

	*U*^11^	*U*^22^	*U*^33^	*U*^12^	*U*^13^	*U*^23^
O1a	0.0310 (8)	0.0231 (8)	0.0286 (8)	0.0104 (6)	−0.0055 (6)	0.0001 (6)
P2a	0.0256 (3)	0.0229 (3)	0.0193 (3)	0.0072 (2)	−0.0004 (2)	0.0017 (2)
S3a	0.0313 (3)	0.0222 (3)	0.0255 (3)	0.0100 (2)	−0.0008 (2)	0.0022 (2)
C4a	0.0276 (11)	0.0264 (12)	0.0311 (12)	0.0065 (9)	0.0054 (9)	0.0046 (10)
C5a	0.0270 (11)	0.0287 (12)	0.0253 (11)	0.0116 (9)	0.0051 (9)	0.0018 (9)
C6a	0.0251 (11)	0.0292 (12)	0.0224 (11)	0.0127 (9)	−0.0002 (8)	−0.0014 (9)
C7a	0.0344 (12)	0.0346 (13)	0.0268 (12)	0.0186 (10)	0.0044 (9)	0.0052 (10)
C8a	0.0458 (15)	0.0510 (16)	0.0269 (12)	0.0304 (13)	−0.0016 (10)	0.0036 (11)
C9a	0.0369 (14)	0.0535 (17)	0.0358 (14)	0.0220 (13)	−0.0118 (11)	−0.0099 (12)
C10a	0.0275 (12)	0.0388 (14)	0.0406 (14)	0.0112 (11)	−0.0020 (10)	−0.0054 (11)
S21a	0.0317 (3)	0.0305 (3)	0.0249 (3)	0.0086 (2)	0.0063 (2)	0.0038 (2)
C11a	0.0255 (11)	0.0197 (11)	0.0224 (11)	0.0048 (9)	0.0014 (8)	0.0024 (8)
C12a	0.0247 (11)	0.0280 (12)	0.0248 (11)	0.0074 (9)	0.0044 (9)	0.0031 (9)
C13a	0.0252 (11)	0.0291 (12)	0.0229 (11)	0.0067 (9)	−0.0011 (9)	0.0024 (9)
C14a	0.0317 (12)	0.0218 (11)	0.0212 (11)	0.0072 (9)	0.0031 (9)	0.0028 (9)
C15a	0.0253 (11)	0.0294 (12)	0.0273 (12)	0.0095 (9)	0.0071 (9)	0.0027 (9)
C16a	0.0252 (11)	0.0258 (11)	0.0283 (12)	0.0094 (9)	−0.0013 (9)	−0.0001 (9)
O14a	0.0348 (9)	0.0405 (9)	0.0191 (8)	0.0126 (7)	0.0020 (6)	−0.0009 (7)
C17a	0.0446 (16)	0.082 (2)	0.0207 (12)	0.0191 (15)	−0.0041 (11)	−0.0052 (13)
O1b	0.0264 (8)	0.0223 (8)	0.0302 (8)	0.0125 (6)	0.0061 (6)	0.0094 (6)
P2b	0.0266 (3)	0.0201 (3)	0.0214 (3)	0.0109 (2)	0.0028 (2)	0.0049 (2)
S3b	0.0268 (3)	0.0240 (3)	0.0277 (3)	0.0084 (2)	0.0004 (2)	0.0021 (2)
C4b	0.0283 (11)	0.0286 (12)	0.0291 (12)	0.0138 (10)	0.0048 (9)	0.0033 (9)
C5b	0.0305 (11)	0.0251 (11)	0.0173 (10)	0.0129 (9)	−0.0013 (8)	0.0019 (8)
C6b	0.0290 (11)	0.0223 (11)	0.0212 (10)	0.0145 (9)	0.0000 (8)	0.0037 (9)
C7b	0.0316 (12)	0.0265 (12)	0.0265 (11)	0.0124 (10)	0.0038 (9)	0.0070 (9)
C8b	0.0459 (14)	0.0238 (12)	0.0294 (12)	0.0140 (11)	0.0018 (10)	0.0071 (10)
C9b	0.0453 (14)	0.0281 (13)	0.0288 (12)	0.0224 (11)	−0.0026 (10)	0.0021 (10)
C10b	0.0333 (12)	0.0318 (13)	0.0233 (11)	0.0178 (10)	−0.0018 (9)	−0.0001 (9)
S21b	0.0435 (3)	0.0294 (3)	0.0286 (3)	0.0226 (3)	0.0073 (2)	0.0039 (2)
C11b	0.0215 (10)	0.0252 (11)	0.0229 (11)	0.0109 (9)	0.0019 (8)	0.0040 (9)
C12b	0.0336 (12)	0.0254 (12)	0.0272 (11)	0.0142 (10)	0.0031 (9)	0.0022 (9)
C13b	0.0326 (12)	0.0274 (12)	0.0254 (11)	0.0138 (10)	0.0031 (9)	0.0076 (9)
C14b	0.0236 (11)	0.0317 (12)	0.0214 (11)	0.0115 (9)	0.0014 (8)	0.0041 (9)
C15b	0.0303 (12)	0.0249 (12)	0.0294 (12)	0.0098 (9)	−0.0016 (9)	−0.0015 (9)
C16b	0.0317 (12)	0.0216 (11)	0.0268 (11)	0.0098 (9)	0.0013 (9)	0.0060 (9)
O14b	0.0442 (9)	0.0359 (9)	0.0215 (8)	0.0169 (8)	0.0004 (7)	0.0037 (7)
C17b	0.0404 (14)	0.0466 (15)	0.0242 (12)	0.0245 (12)	0.0035 (10)	0.0081 (11)

### Geometric parameters (Å, °)

**Table:**

O1a—C6a	1.404 (3)	O1b—C6b	1.412 (2)
O1a—P2a	1.6119 (15)	O1b—P2b	1.6033 (15)
P2a—C11a	1.794 (2)	P2b—C11b	1.796 (2)
P2a—S21a	1.9219 (8)	P2b—S21b	1.9197 (7)
P2a—S3a	2.0766 (8)	P2b—S3b	2.0586 (8)
S3a—C4a	1.835 (2)	S3b—C4b	1.831 (2)
C4a—C5a	1.497 (3)	C4b—C5b	1.505 (3)
C4a—H4a1	0.9900	C4b—H4b1	0.9900
C4a—H4a2	0.9900	C4b—H4b2	0.9900
C5a—C6a	1.385 (3)	C5b—C6b	1.385 (3)
C5a—C10a	1.396 (3)	C5b—C10b	1.401 (3)
C6a—C7a	1.375 (3)	C6b—C7b	1.386 (3)
C7a—C8a	1.392 (3)	C7b—C8b	1.385 (3)
C7a—H7a	0.9500	C7b—H7b	0.9500
C8a—C9a	1.375 (4)	C8b—C9b	1.380 (3)
C8a—H8a	0.9500	C8b—H8b	0.9500
C9a—C10a	1.386 (4)	C9b—C10b	1.377 (3)
C9a—H9a	0.9500	C9b—H9b	0.9500
C10a—H10a	0.9500	C10b—H10b	0.9500
C11a—C12a	1.389 (3)	C11b—C12b	1.392 (3)
C11a—C16a	1.398 (3)	C11b—C16b	1.396 (3)
C12a—C13a	1.385 (3)	C12b—C13b	1.386 (3)
C12a—H12a	0.9500	C12b—H12b	0.9500
C13a—C14a	1.393 (3)	C13b—C14b	1.390 (3)
C13a—H13a	0.9500	C13b—H13b	0.9500
C14a—O14a	1.355 (3)	C14b—O14b	1.365 (3)
C14a—C15a	1.393 (3)	C14b—C15b	1.384 (3)
C15a—C16a	1.378 (3)	C15b—C16b	1.380 (3)
C15a—H15a	0.9500	C15b—H15b	0.9500
C16a—H16a	0.9500	C16b—H16b	0.9500
O14a—C17a	1.428 (3)	O14b—C17b	1.434 (3)
C17a—H17a	0.9800	C17b—H17d	0.9800
C17a—H17b	0.9800	C17b—H17e	0.9800
C17a—H17c	0.9800	C17b—H17f	0.9800
			
C6a—O1a—P2a	124.10 (13)	C6b—O1b—P2b	127.23 (13)
O1a—P2a—C11a	99.46 (9)	O1b—P2b—C11b	104.09 (9)
O1a—P2a—S21a	118.15 (7)	O1b—P2b—S21b	112.57 (6)
C11a—P2a—S21a	114.65 (7)	C11b—P2b—S21b	114.84 (7)
O1a—P2a—S3a	103.99 (6)	O1b—P2b—S3b	104.42 (6)
C11a—P2a—S3a	109.24 (7)	C11b—P2b—S3b	108.63 (7)
S21a—P2a—S3a	110.34 (3)	S21b—P2b—S3b	111.54 (3)
C4a—S3a—P2a	98.13 (8)	C4b—S3b—P2b	95.79 (8)
C5a—C4a—S3a	109.77 (15)	C5b—C4b—S3b	113.98 (15)
C5a—C4a—H4a1	109.7	C5b—C4b—H4b1	108.8
S3a—C4a—H4a1	109.7	S3b—C4b—H4b1	108.8
C5a—C4a—H4a2	109.7	C5b—C4b—H4b2	108.8
S3a—C4a—H4a2	109.7	S3b—C4b—H4b2	108.8
H4a1—C4a—H4a2	108.2	H4b1—C4b—H4b2	107.7
C6a—C5a—C10a	117.8 (2)	C6b—C5b—C10b	116.7 (2)
C6a—C5a—C4a	119.70 (19)	C6b—C5b—C4b	124.61 (18)
C10a—C5a—C4a	122.5 (2)	C10b—C5b—C4b	118.6 (2)
C7a—C6a—C5a	123.1 (2)	C5b—C6b—C7b	122.63 (19)
C7a—C6a—O1a	118.32 (19)	C5b—C6b—O1b	123.69 (19)
C5a—C6a—O1a	118.51 (19)	C7b—C6b—O1b	113.68 (18)
C6a—C7a—C8a	118.1 (2)	C8b—C7b—C6b	118.9 (2)
C6a—C7a—H7a	121.0	C8b—C7b—H7b	120.6
C8a—C7a—H7a	121.0	C6b—C7b—H7b	120.6
C9a—C8a—C7a	120.3 (2)	C9b—C8b—C7b	120.1 (2)
C9a—C8a—H8a	119.8	C9b—C8b—H8b	119.9
C7a—C8a—H8a	119.8	C7b—C8b—H8b	119.9
C8a—C9a—C10a	120.8 (2)	C10b—C9b—C8b	120.0 (2)
C8a—C9a—H9a	119.6	C10b—C9b—H9b	120.0
C10a—C9a—H9a	119.6	C8b—C9b—H9b	120.0
C9a—C10a—C5a	120.0 (2)	C9b—C10b—C5b	121.6 (2)
C9a—C10a—H10a	120.0	C9b—C10b—H10b	119.2
C5a—C10a—H10a	120.0	C5b—C10b—H10b	119.2
C12a—C11a—C16a	118.58 (19)	C12b—C11b—C16b	119.02 (19)
C12a—C11a—P2a	119.61 (16)	C12b—C11b—P2b	118.69 (16)
C16a—C11a—P2a	121.64 (16)	C16b—C11b—P2b	122.27 (16)
C13a—C12a—C11a	121.5 (2)	C13b—C12b—C11b	121.0 (2)
C13a—C12a—H12a	119.2	C13b—C12b—H12b	119.5
C11a—C12a—H12a	119.2	C11b—C12b—H12b	119.5
C12a—C13a—C14a	119.1 (2)	C12b—C13b—C14b	119.1 (2)
C12a—C13a—H13a	120.5	C12b—C13b—H13b	120.4
C14a—C13a—H13a	120.5	C14b—C13b—H13b	120.4
O14a—C14a—C13a	124.18 (19)	O14b—C14b—C15b	115.25 (19)
O14a—C14a—C15a	115.77 (19)	O14b—C14b—C13b	124.3 (2)
C13a—C14a—C15a	120.05 (19)	C15b—C14b—C13b	120.4 (2)
C16a—C15a—C14a	120.14 (19)	C16b—C15b—C14b	120.3 (2)
C16a—C15a—H15a	119.9	C16b—C15b—H15b	119.9
C14a—C15a—H15a	119.9	C14b—C15b—H15b	119.9
C15a—C16a—C11a	120.6 (2)	C15b—C16b—C11b	120.2 (2)
C15a—C16a—H16a	119.7	C15b—C16b—H16b	119.9
C11a—C16a—H16a	119.7	C11b—C16b—H16b	119.9
C14a—O14a—C17a	117.98 (18)	C14b—O14b—C17b	117.99 (17)
O14a—C17a—H17a	109.5	O14b—C17b—H17d	109.5
O14a—C17a—H17b	109.5	O14b—C17b—H17e	109.5
H17a—C17a—H17b	109.5	H17d—C17b—H17e	109.5
O14a—C17a—H17c	109.5	O14b—C17b—H17f	109.5
H17a—C17a—H17c	109.5	H17d—C17b—H17f	109.5
H17b—C17a—H17c	109.5	H17e—C17b—H17f	109.5
			
C6a—O1a—P2a—C11a	−146.02 (17)	C6b—O1b—P2b—C11b	−82.72 (17)
C6a—O1a—P2a—S21a	89.32 (17)	C6b—O1b—P2b—S21b	152.30 (14)
C6a—O1a—P2a—S3a	−33.33 (17)	C6b—O1b—P2b—S3b	31.14 (17)
O1a—P2a—S3a—C4a	−19.97 (10)	O1b—P2b—S3b—C4b	−50.90 (9)
C11a—P2a—S3a—C4a	85.47 (11)	C11b—P2b—S3b—C4b	59.70 (10)
S21a—P2a—S3a—C4a	−147.62 (8)	S21b—P2b—S3b—C4b	−172.74 (8)
P2a—S3a—C4a—C5a	59.70 (15)	P2b—S3b—C4b—C5b	55.70 (16)
S3a—C4a—C5a—C6a	−55.1 (2)	S3b—C4b—C5b—C6b	−35.7 (3)
S3a—C4a—C5a—C10a	124.4 (2)	S3b—C4b—C5b—C10b	145.92 (17)
C10a—C5a—C6a—C7a	0.1 (3)	C10b—C5b—C6b—C7b	−1.2 (3)
C4a—C5a—C6a—C7a	179.6 (2)	C4b—C5b—C6b—C7b	−179.7 (2)
C10a—C5a—C6a—O1a	176.18 (19)	C10b—C5b—C6b—O1b	178.88 (18)
C4a—C5a—C6a—O1a	−4.2 (3)	C4b—C5b—C6b—O1b	0.4 (3)
P2a—O1a—C6a—C7a	−128.39 (18)	P2b—O1b—C6b—C5b	−1.6 (3)
P2a—O1a—C6a—C5a	55.3 (2)	P2b—O1b—C6b—C7b	178.51 (15)
C5a—C6a—C7a—C8a	0.6 (3)	C5b—C6b—C7b—C8b	1.8 (3)
O1a—C6a—C7a—C8a	−175.56 (19)	O1b—C6b—C7b—C8b	−178.33 (18)
C6a—C7a—C8a—C9a	−0.8 (3)	C6b—C7b—C8b—C9b	−0.6 (3)
C7a—C8a—C9a—C10a	0.4 (4)	C7b—C8b—C9b—C10b	−0.9 (3)
C8a—C9a—C10a—C5a	0.3 (4)	C8b—C9b—C10b—C5b	1.5 (3)
C6a—C5a—C10a—C9a	−0.5 (3)	C6b—C5b—C10b—C9b	−0.4 (3)
C4a—C5a—C10a—C9a	180.0 (2)	C4b—C5b—C10b—C9b	178.1 (2)
O1a—P2a—C11a—C12a	−150.77 (17)	O1b—P2b—C11b—C12b	−164.91 (16)
S21a—P2a—C11a—C12a	−23.7 (2)	S21b—P2b—C11b—C12b	−41.39 (19)
S3a—P2a—C11a—C12a	100.70 (17)	S3b—P2b—C11b—C12b	84.27 (17)
O1a—P2a—C11a—C16a	33.9 (2)	O1b—P2b—C11b—C16b	16.9 (2)
S21a—P2a—C11a—C16a	160.92 (16)	S21b—P2b—C11b—C16b	140.42 (16)
S3a—P2a—C11a—C16a	−74.66 (19)	S3b—P2b—C11b—C16b	−93.92 (18)
C16a—C11a—C12a—C13a	0.6 (3)	C16b—C11b—C12b—C13b	1.1 (3)
P2a—C11a—C12a—C13a	−174.86 (17)	P2b—C11b—C12b—C13b	−177.12 (17)
C11a—C12a—C13a—C14a	0.3 (3)	C11b—C12b—C13b—C14b	−0.2 (3)
C12a—C13a—C14a—O14a	179.2 (2)	C12b—C13b—C14b—O14b	178.4 (2)
C12a—C13a—C14a—C15a	−1.1 (3)	C12b—C13b—C14b—C15b	−1.1 (3)
O14a—C14a—C15a—C16a	−179.2 (2)	O14b—C14b—C15b—C16b	−178.10 (19)
C13a—C14a—C15a—C16a	1.1 (3)	C13b—C14b—C15b—C16b	1.4 (3)
C14a—C15a—C16a—C11a	−0.1 (3)	C14b—C15b—C16b—C11b	−0.4 (3)
C12a—C11a—C16a—C15a	−0.7 (3)	C12b—C11b—C16b—C15b	−0.8 (3)
P2a—C11a—C16a—C15a	174.71 (17)	P2b—C11b—C16b—C15b	177.37 (17)
C13a—C14a—O14a—C17a	−0.6 (3)	C15b—C14b—O14b—C17b	179.73 (19)
C15a—C14a—O14a—C17a	179.7 (2)	C13b—C14b—O14b—C17b	0.2 (3)
